# Effects of soil moisture on ^13^C assimilate redistribution and grain yield components in wheat

**DOI:** 10.3389/fpls.2025.1527224

**Published:** 2025-05-26

**Authors:** Zhen Zhang, Zhenwen Yu, Yu Shi, Yongli Zhang

**Affiliations:** Key Laboratory of Crop Physiology, Ecology and Farming, Ministry of Agriculture, Shandong Agricultural University, Taian, Shandong, China

**Keywords:** micro-sprinkler, water-saving irrigation, 13 C assimilate redistribution, endogenous hormone, grain yield

## Abstract

**Introduction:**

In order to solve the current situation of water shortage and achieve sustainable agricultural development, micro-sprinkler water-saving irrigation is one of the effective methods to improve water use efficiency (WUE) compared with flood irrigation. However, the effects of water content on wheat grain weight and plant hormone content under micro-sprinkler water-saving irrigation, and the potential mechanism of different water content on plant hormone-mediated grain grouting under micro-sprinkler water-saving irrigation are still largely unknown.

**Methods:**

Therefore, this study conducted extensive monitoring of wheat grain weight and plant hormone content under different water content in a typical winter wheat field (wheat) in the North China Plain from 2019 to 2021 by 13C isotope tracer technology through a field experiment based on micro-sprinkling water-saving irrigation.

**Results:**

The results showed that under micro-sprinkler water saving irrigation, the lateral development of wheat roots after anthesis was promoted by W3 treatment in the deep soil depth (0-60 cm), which was the basis for efficient absorption of water and fertilizer, as well as efficient formation of photosynthate. Meanwhile, W3 treatment significantly promoted the transfer of photosynthetic products from leaves, stems and sheaths to grain. Compared with other treatments, W3 treatment significantly increased the average grain filling rate and grain filling time. Compared with W1, W2 and W5 treatments, W3 and W4 treatments significantly improved the number of grains per ear, 1000 grain weight and grain yield. From the perspective of water saving, W3 treatment had the highest effect. Compared with W1, W2 and W5 treatments, W3 treatment significantly increased the average grain yield of the two seasons by 19.69%, 6.30% and 8.07%, respectively.

**Discussion:**

In this study, optimizing micro-sprinkler water saving irrigation can improve root development, promote photosynthetic product transport, and increase average grain filling rate and grain filling time, thereby increasing grain yield.This study provides valuable insights into improving sustainable wheat production in micro-water-saving irrigation agricultural cropping systems, and it may provide a practical framework for striking a balance between groundwater protection and food security.

## 1 Introduction

The North China Plain (NCP) is the primary food-producing region, and provides about 61% of the nation’s wheat with less than 8% of the total water resources in China (Liu et al., 2022). However, the annual rainfall is uneven and mainly concentrates in the summer maize season, while only 20%-30% falls in the winter wheat growing season, which meets only 25%-40% of winter wheat requirements (Kang et al., 2017). Meanwhile, water shortages are becoming increasingly serious. To achieve expected wheat grain yield, farmers in NCP usually irrigate three to five times via flood irrigation throughout the wheat growing season. This irrigation regime improves grain yield, but reduces water use efficiency due to supplying too much water. The extensive use of groundwater for irrigation has results in groundwater table dropping continuously, compromising both hydrological balance and sustainable agricultural production (Sun et al., 2015). Therefore, deficit irrigation is critical for maintaining high wheat production, and an optimal irrigation water management scheme must be developed for ecological security and sustainable development of winter wheat production in this region.

Compared with traditional flood irrigation, micro-sprinkler water-saving irrigation affected the infiltration and redistribution of water and fertilizer in soil, resulting in the temporal and spatial distribution differences of water and nutrients. As a result, soil physiological and biochemical characteristics will change significantly, which will affect the wheat root growth, development and metabolism, and affect the aboveground plant through the root system (Li et al., 2019). Therefore, understanding root system’s adaptive responses specific to soil environments will provide important opportunities to reveal the mechanism of micro-sprinkler water-saving irrigation to promote efficient utilization of water. However, the current studies on water-saving irrigation mainly focus on the water-saving effects, irrigation system selection, and soil nutrient migration, etc (Wan et al., 2022), and the researches on the physiological response of root system to micro-sprinkler water-saving irrigation and its regulation effect on the grain yield components is relatively insufficient.

Grain filling is a pivotal stage for yield components in wheat. Formation and redistribution of photosynthates after anthesis is the major component of wheat grain, which comprises 80% of the dry weight of wheat grain (Yang and Zhang, 2010). In general, pre-anthesis assimilate in wheat vegetative organs contributes to 20%-35% of the grain weight. Under water stress, the contribution increased up to 80%. Water stress during early grain-filling curtails the grain sink potential by reducing both the rate and duration of grain filling, but increases the remobilization of carbon reserves in stem to grains. These results suggest that promote grain filling could be an effective way to grain yield.

The plant hormones, which are minor chemicals in wheat, play critical roles in regulating plant growth and stress responses (Yu et al., 2020). It has been well demonstrated that the hormones are involved in regulating grain filling in wheat (Panda et al., 2018). The abscisic acid (ABA) content in wheat grains was found to be significantly correlated with grain-filling rate (Wang et al., 2015). In addition, ABA content was found to be significantly higher in superior spikelets than in inferior spikelets (Nonhebel and Griffin, 2020). Moderate soil water deficit can increase the ABA level in inferior spikelets, which thus promotes grain filling of inferior spikelets (Teng et al., 2021). Furthermore, the indole acetic acid (IAA) content was much higher in superior spikelets than in inferior spikelets during grain filling in wheat (Chang et al., 2020; Nonhebel and Griffin, 2020). These studies suggested that the plant hormones content were of great importance in regulating both panicle development and grain filling.

Plant hormones were well demonstrated to play key roles in regulating wheat grain filling, especially in promoting the middle and last grain filling (Teng et al., 2021). However, whether plant hormones are involved in regulating grain filling in response to different micro-sprinkler water-saving irrigation is still ambiguous. The purpose of this study was to investigate the functions of plant hormones in regulating grain filling at the grain-filling process under different micro-sprinkler water-saving irrigation conditions. A two-year field experiment was conducted to reveal the effects of micro-sprinkler water-saving irrigation on wheat grain weight and plant hormone contents, as well as the underlying mechanisms of plant hormone-mediated grain filling under micro-sprinkler water-saving irrigation. The outcomes of this study provide useful information for wheat production to achieve higher grain yield and provide insight into the mechanisms underlying the interactions between irrigation and plant hormones on wheat growth.

## 2 Materials and methods

### 2.1 Experimental site

**Figure 1:**
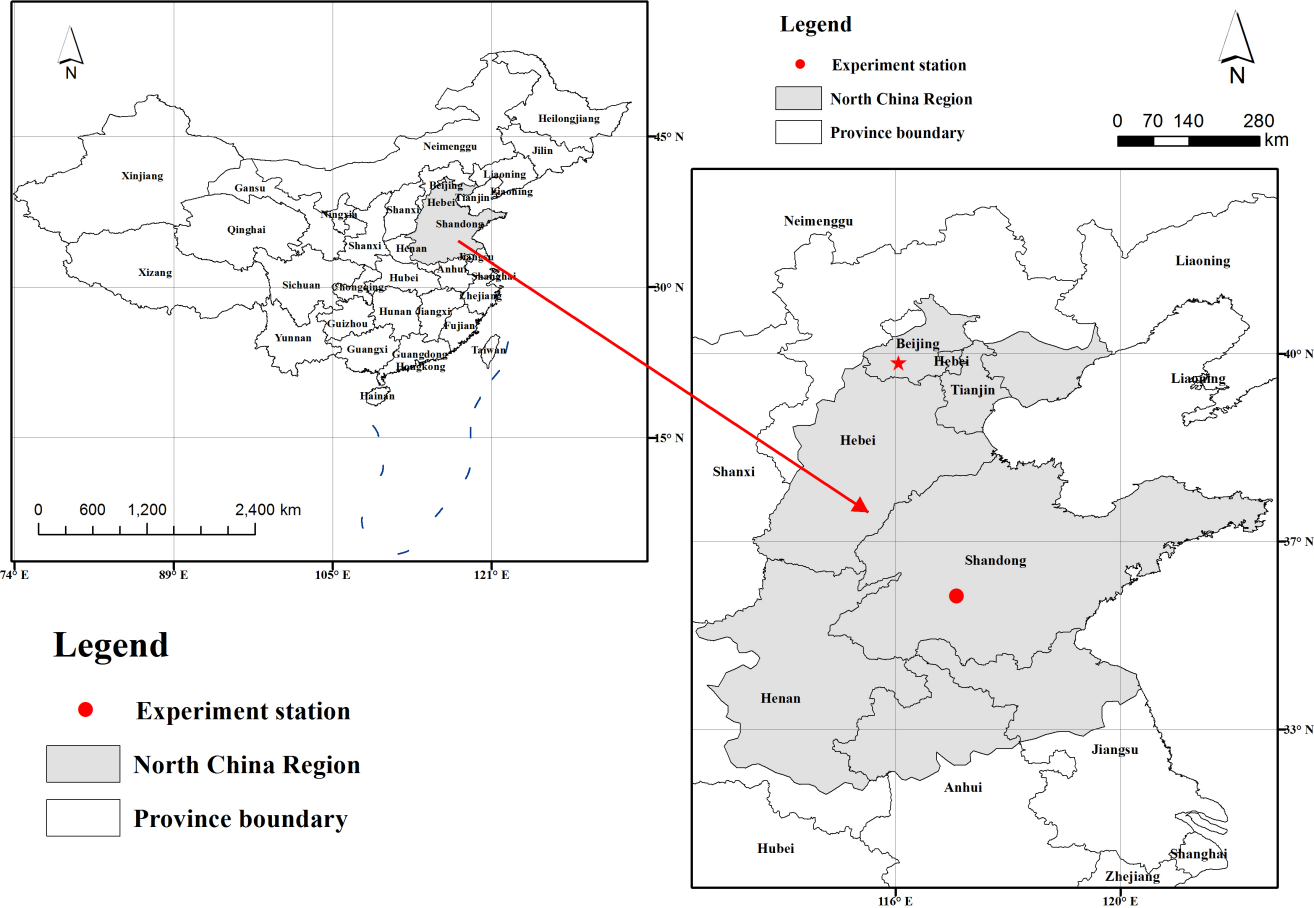
The study site map.

**Figure 2:**
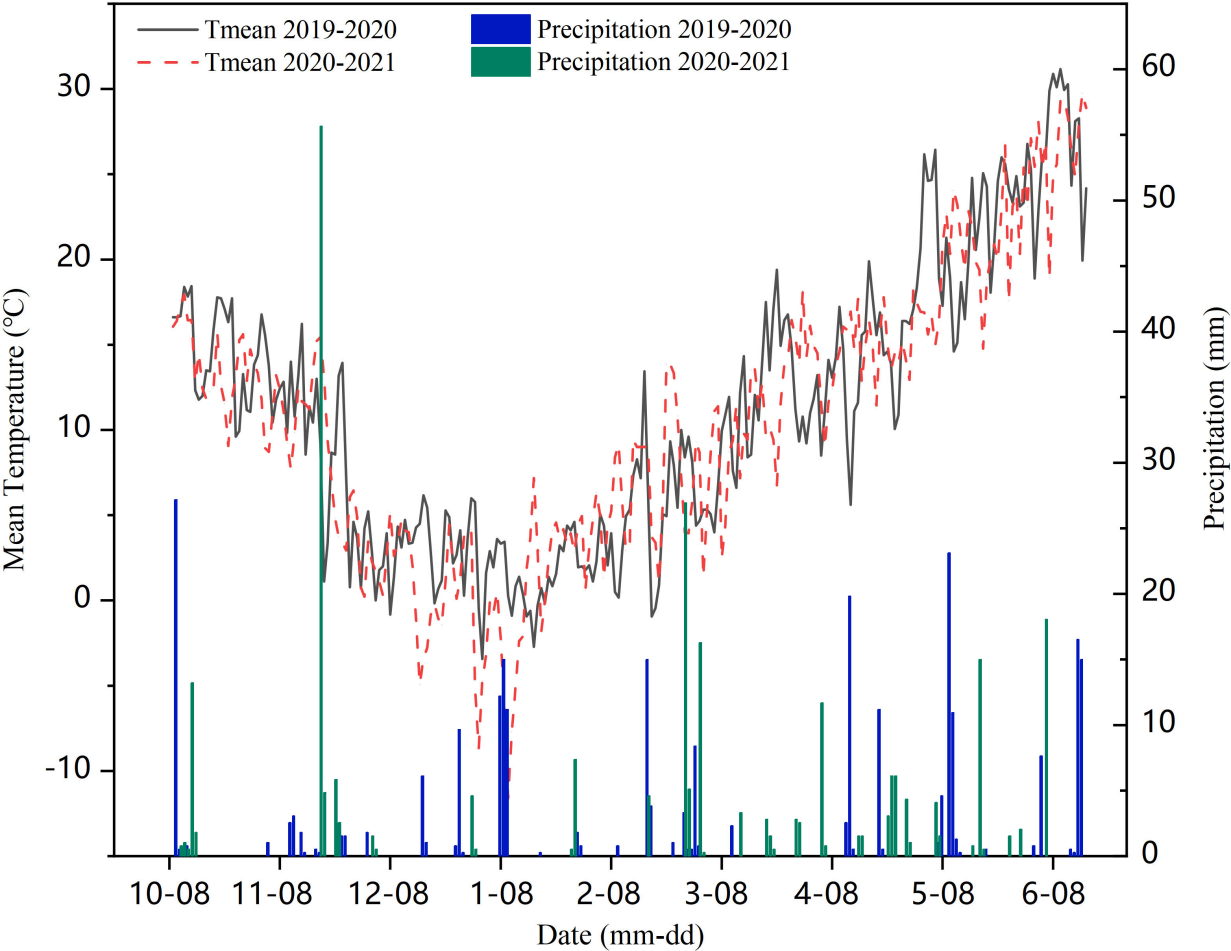
Effective precipitation and temperature during wheat growth period.

In 2019 and 2021, field experiments were conducted in an experimental field (35°40′ N, 116°41′ E) (**Figure 1**) at Yanzhou Academician Work Experimental Station in Shandong Agricultural University, Ji’ning, Shandong Province, China. The site is characterized with temperate monsoon zone climate with mean annual precipitation of 600 mm and average annual temperature of 14.1°C. The meteorological data were obtained from Ji’ning meteorological station. Weather details of experimental duration in both years are given in **Figure 2**. According to the Food and Agriculture Organization of the United Nations classification, the site soil at the experimental site is loam, with the following properties: 14.20 g·kg^-1^ the content of organic carbon, 1.13 g·kg^-1^ total nitrogen, 122.60 mg·kg^-1^ available nitrogen, 129.44 mg·kg^-1^ available potassium, and 38.11 mg·kg^-1^ available phosphorus.

### 2.2 Experimental design

Based on previous studies (Zhang et al., 2023; Man et al., 2014a), we established five irrigation regimes. Five irrigation regimes i.e. wherein supplementary irrigation brought soil water content in the 0~40 cm profile to 65% (W1) field water capacity (FC), 70% (W2) FC, 75% (W3) FC, 80% (W4) FC or 85% (W5) FC at the joining and anthesis stages, were established in split plot arrangement (20 m long and 2 m wide) under randomized complete block design with three replications. There are two irrigation events in the growing season: the first at jointing and the second at anthesis. Soil water content was determined by drying method before irrigation at jointing and anthesis. The amount of supplemental irrigation matched the treatment irrigation requirement, which was calculated from the relative soil moisture content in the 0–140 cm soil depth. Field water capacity is defined as the water content of a soil following saturation with water when free drainage is negligible. The supplemental irrigation amounts were calculated according to the method of Man et al. (2014b). All irrigation treatments were conducted using a micro-sprinkling hose, it’s length was 20 m, and it’s flow rate was 6 m^3^·h^-1^ under the working pressure of 0.02 MPa. The supplemental irrigation amount were controlled by water meter at the head of micro-sprinkling hoses, and the sprinkling angle of hose was 80°. In this study, the micro-sprinkling hoses had folded diameters of 6 cm, and were perforated with 58 groups of orifices. There were six orifices in each group, and with a group, the diameter of the first and the sixth orifices was 1.2 mm, while the diameter of the other four orifices was 0.8 mm. The distance between two adjacent groups of orifices was 20 cm (Man et al., 2014a).

The variety of wheat for the trial was Jimai 22, respectively. The wheat seeded on October 12, 2019 and harvested on June 11, 2020 seeded on October 8, 2020 and harvested on June 13, 2021. The planting density was 1800000 plants per hectare. All treatments had the identical fertilization scheme. The basal fertilizer consisted of single superphosphate and potassium sulphate, providing 150 kg P_2_O_5_ ha^−1^ and 150 kg K_2_O ha^−1^. Nitrogen fertilizer was also applied at 135 kg·ha^−1^ and 105 kg·ha^−1^ as a basal fertilizer and topdressing fertilizer at the seedling and jointings, respectively. Before sowing, the base fertilizer was spread onto the soil surface and immediately mixed with a rotary cultivator to a depth of 20 cm. At jointing, a furrow was opened to apply nitrogen fertilizer, and then covered immediately. Pesticides and herbicides were applied as necessary.

### 2.3 Root weight density and root surface area density

The root samples of 0-20, 20-40, 40-60 cm soil depths were collected in wheat planting rows and between rows with a root drill at anthesis and 10 and 20 days after anthesis. The diameter of the root drill was 10 cm. The mixture of root and soil was put into a 100 mesh nylon bag and washed with tap water. After washing, the mixture of root and organic debris was carefully separated. The separated root samples were stored in a -40°C refrigerator. Place the root sample in an 80 °C oven and dry it to constant weight to measure the dry weight of root system and calculate the dry weight density of root system. The root surface area was measured by WinRHIZO2013 system and EpsonV700 root scanner (Gleason et al., 2019).

### 2.4 Carbon isotope analysis

We conducted a leaf isotope tagging experiment with ^13^CO_2_ in both wheat growing seasons. Ten representative single stem in each wheat cultivation plot were selected at the anthesis stage. We encased the flag leaf of each selected single stem in a 0.1-mm-thick Mylar plastic bag, which permitted sunlight to pass at levels up to 95% of the natural intensity. The bags were sealed at the base with adhesive tape and subsequently injected with 3.5 mL of ^13^CO_2_. After allowing photosynthesis to proceed for 30 min, the ^13^CO_2_ in each bag was extracted through a KOH washer to absorb the remaining ^13^CO_2_ and the bag was removed. This experiment was conducted from 09:00–11:00 a.m. on sunny days. At 72 h after procesing from anthesis and maturity stages, the wheat plants were randomly sampled from each plot by cutting at the ground level. All plants were separated into stems and sheaths, leaves, glumes (spike axis and kernel husks), and grains (only at maturity), and oven-dried to a constant weight at 70 °C to determine the aboveground biomass. All samples were milled to a fine powder using a ball mill, for use in the carbon isotope analysis. The carbon isotope content of milled samples (5 mg) was determined using an online system composed of an elemental analyzer, a TripleTrap, and a mass spectrometer(Carlo Erba 2100, Milan, Italy). The distribution of ^13^C photosynthates among different organs was determined (Gao et al., 2017). The formula for calculating relevant indicators is as follows:


$${\text{δ}}^{13}\text{C}(\%)=(\text{Rs}/\text{R}-1)\times 100$$

Where Rs is ^13^C/^12^C in the measured sample, and R is a fixed value of 0.0112372.


$$\text{CA}(\%)=[({\text{δ}}^{13}\text{C}+1000)\times \text{R}\times 100]/[({\text{δ}}^{13}\text{C}+1000)\times \text{R}+100]$$

Where CA is ^13^C abundance.


CD(ug)=(CAL−CAU)/100×OW×CCO×1000

Where CD is ^13^C distribution for each organ, CAL is ^13^C abundance in labeled sample, CAU is ^13^C abundance in un-labeled sample, OW is organ weight, CCO is carbon content of organ.


$$\text{CT}(\text{ug})={\text{CAAL}}_{72}-\text{CAAM}.$$

Where CT is ^13^C transhipment, CAAL_72_ is ^13^C accumulation amount after label 72 h, CAAM is ^13^C accumulation amount at maturity.

### 2.5 Determination of endogenous hormones in seeds

The root of 0-20, 20-40, 40-60 cm soil depths were sampled at anthesis and 10 and 20 days after anthesis, and immersed in liquid nitrogen and then maintained in a -80°C freezer for measurement of endogenous hormones. Hormones were determined by liquid chromatography-mass spectrometry (LC-MS/MS). First, the hormone components in the sample are separated by liquid chromatography, and then the separated components are qualitatively and quantitatively analyzed by mass spectrometry. Operation process, sample pretreatment: Weigh an appropriate amount of plant samples, such as rice seedlings, extract with 80% methanol at 4°C for 12h after liquid nitrogen grinding, collect the supernatant by centrifugation, blow dry with nitrogen, redissolve with 95% acetonitrile, and then centrifuge the supernatant for LC-MS/MS analysis.

Instrumental analysis: Appropriate chromatographic column and mobile phase system, such as Waters Acquity UPLC HSS T3 C18 column, water (0.1% acetic acid) -acetonitrile (0.1% acetic acid) as the mobile phase for depth elution. Mass spectrometry uses electrospray ion source, detected in positive ion mode, and selected reaction monitoring (MRM) mode to monitor specific ion pairs of each hormone.

### 2.6 Grain weight and grain filling

The grain sample were taken at 7, 14, 21, 28, 35 days after anthesis in both growth season. The formula for calculating grain filling rate (G) is provided below (Wu et al., 2020).


$$\text{G}(\text{g}\cdot {\text{d}}^{-1})=\Delta \text{W}/\Delta \text{d}$$

Where ΔW is the difference in 1000-grain weight between two sample time points, and Δd is the number of days between two sample time points, and Δd in this experiment is 7.

The grain-filling process was fitted by logistic growth equation as described by Liu et al. (2021).


$$\text{G}=\frac{\text{A}k\text{B}e^{-\text{kt}}}{N(1+\text{B}e^{-\text{kt}})(\frac{N+1}{N})}$$

Where kernel weight (mg), A is final kernel weight (mg), t is time after anthesis (d), B, k and N is coefficients determined by regression.

### 2.7 Grain yield, yield components, and harvest index

At maturity, a sample of 4 m^2^ sampling plot was harvested by hand to estimate the yield and spike number per plot. Threety representative plants were chosen to measure the grain number per spike. Subsequently, the grain yield was calculated at 12.5% water content. The 1000-grain weight was calculated by weighing 1000 grains.

### 2.8 Statistical analysis

A statistical analysis software (SPSS 12.5, Chicago, IL, USA) was used for data analysis. The mean values for different treatments were compared using Duncan’s multiple-range test at a probability level of 0.05.

## 3 Results

### 3.1 Root dry weight density and root surface area density

**Figure 3:**
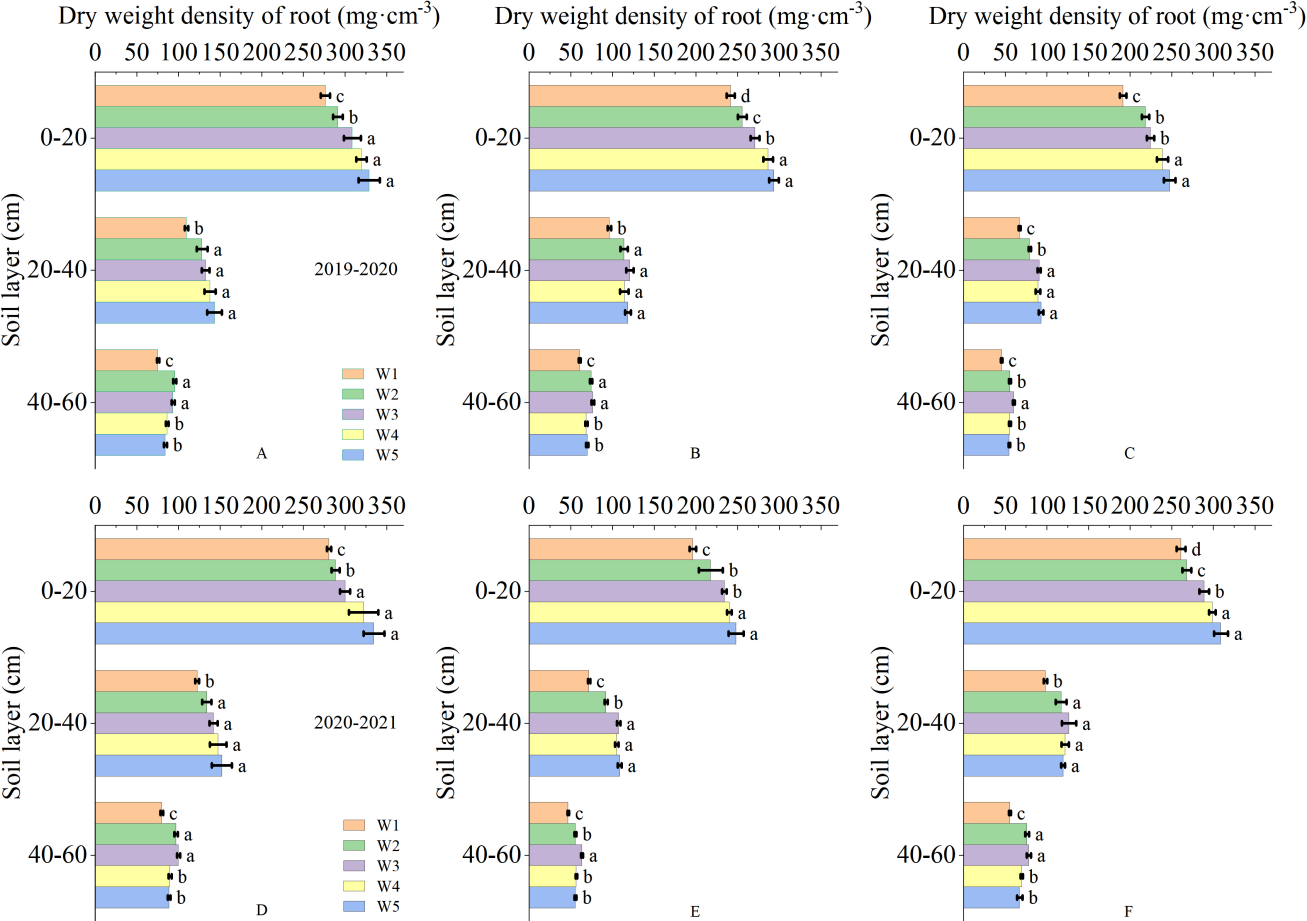
Dry weight density of root in 0-60 cm soil depth after anthesis under different treatments (mg·cm^-3^). **(A, D)** are the anthesis, **(B, E)** are the 10 after anthesis, **(C, F)** are the 20 days after anthesis. Lowercase letters represent that the data difference between processing is greater than 0.05.

**Figure 4:**
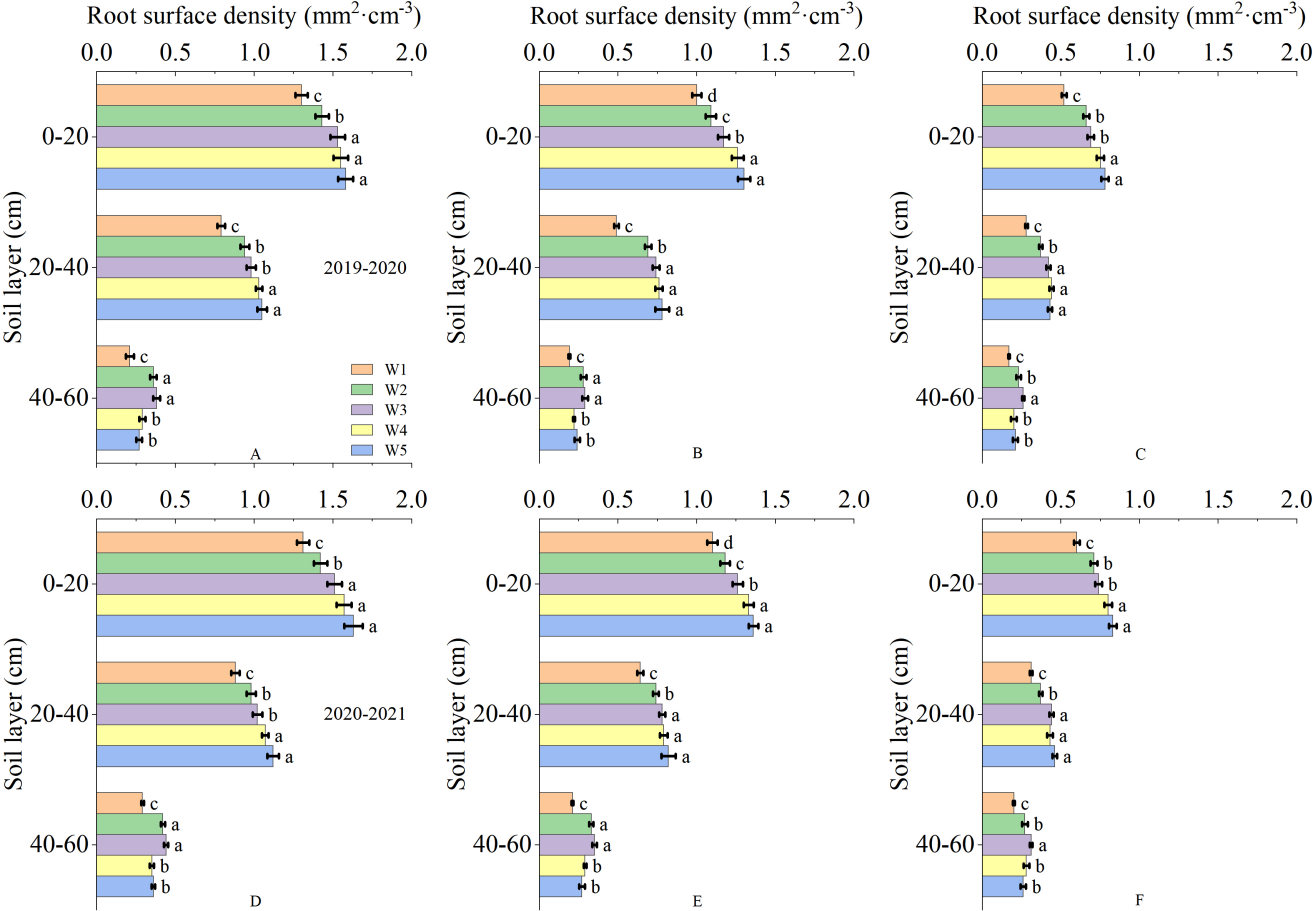
Root surface area density in 0-60 cm soil depth after anthesis under different treatments (mm^2^·cm^-3^). **(A, D)** are the anthesis, **(B, E)** are the 10 after anthesis, **(C, F)** are the 20 days after anthesis. Lowercase letters represent that the data difference between processing is greater than 0.05.

The root in the soil was primarily distributed in the 0-60 cm soil depth. As the depth of the soil depth increased, the root dry weight density and root surface area density gradually deceased (**Figure 3**). The irrigation levels had significant effects on the root dry weight density and root surface area density at anthesis, 10 and 20 days after anthesis. At anthesis stage and 10 days after anthesis, the root dry weight density of 0-20 cm soil layer in W3 treatment was significantly higher than that in W1 and W2 treatment, but significantly lower than that in W4 and W5 treatment. The root dry weight density of 20-40 cm soil layer showed that W3 treatment was significantly higher than W1 treatment, but there was no significant difference between W2, W4 and W5 treatment. The root dry weight density of 40-60 cm soil layer showed no significant difference between W3 treatment and W2 treatment, but was significantly higher than other treatments. At 20 days after anthesis, the root dry weight density of 0-20 cm soil layer showed that W3 treatment was significantly higher than W1 treatment, but significantly lower than W4 and W5 treatment, and the root dry weight density of 20-40 cm soil layer showed that W3 treatment was significantly higher than W1 and W2 treatment, but had no significant difference from W4 and W5 treatment. The root dry weight density of 40-60 cm soil layer in W3 treatment was significantly higher than that in other treatments. The root surface area density and root dry weight density showed a similar trend (**Figure 4**). This results indicated that the W3 treatment improved the root distribution, which was conducive to increase the absorption of soil water and nutrients.

### 3.2 ^13^C photosynthate distribution in different organs of wheat

The amount and ratio of C allocation into various organs were calculated based on the variation in N content and ^13^C isotope in different organs. In 2019-2020, the ^13^C photosynthate in leaf, stem and sheath of W3 treatment were significantly higher than that of W1 treatment after 72 h labeling, whereas we detected no significant differences between W2, W3, W4 and W5 treatments. No significant differences in spike axis and husk ^13^C photosynthate were observed between treatments 72 h after labeling. The ^13^C photosynthate in leaf of W3 treatment was significantly lower than that of W4 and W5 treatments, and there was no significant difference between W1, W2 and W3 treatments. The ^13^C photosynthate in stem and sheath of W3 treatment was significantly lower than that of W5 treatment, and significantly higher than that of W1, W2 treatment, there was no significant difference between W4 treatment. The ^13^C photosynthate in spike axis and husk of W3 treatment was significantly lower than that of W5 treatment, and there was no significant difference between W1, W2, W3 and W4 treatments. The ^13^C photosynthate in grain under the W3 treatment was higher by an of 20.78%, 7.21% and 8.66% than W1, W2 and W5, respectively, there was no significant difference between W4 treatment. Similar responses to treatments were observed in both growth seasons. This indicated that the W3 treatment significantly increased the assimilation of carbohydrate after anthsis and for the distribution of those carbohydrate from vegetative organs to grains. This was probably a key reason for the W3 treatment effect of high grain weight. The differences in the allocation of 72 hleaf, Stem and sheath, Spike axis and husk ^13^C after marking were extremely significant between treatments and between years, while the differences in the allocation of Year×Treatments were not significant. The differences in the allocation of leaf, Spike axis and husk and Grain ^13^C between treatments and between years were extremely significant, and the differences in the allocation of Stem and sheath ^13^C between treatments were significant, and the differences between years were extremely significant. Year×Treatments were significantly different in leaf at maturity, Spike axis and husk ^13^C were extremely significant, and the differences in the allocation of Stem and sheath and Grain ^13^C were not significant.

### 3.3 ^13^C Photosynthate translocation after anthesis

**Figure 5:**
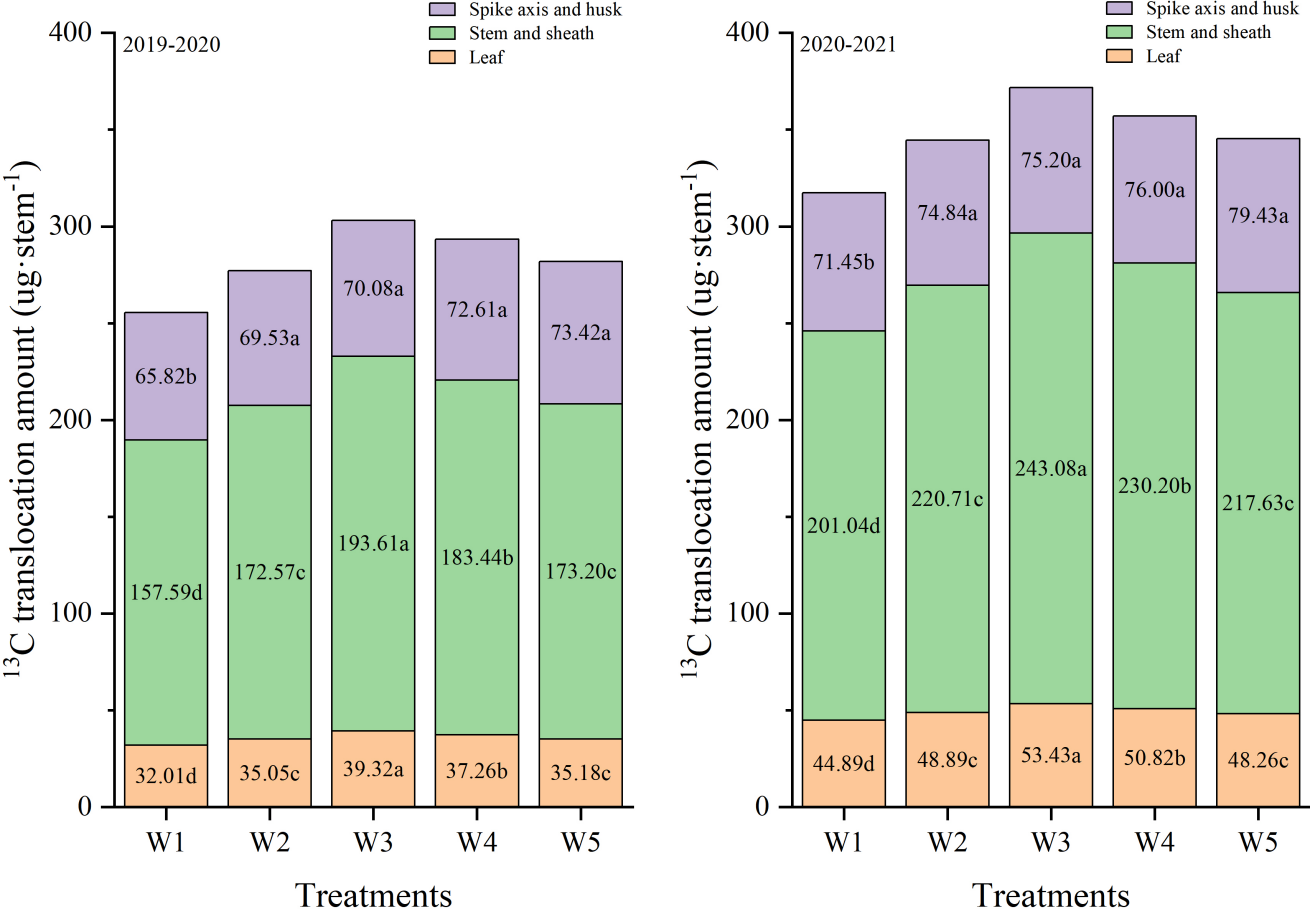
Effects of different treatments on ^13^C translocation amount in different organs at 72h after labeling and maturity in wheat plants.

Changes in the amount of ^13^C distribution in different organs after anthesis are shown in **Figure 5**. In both growth seasons, irrigation treatments had significantly effect on the translocation amount of different organs. W3 treatment significantly improved the transport of leaf, stem and sheath ^13^C photosynthate to grains (**Table 1**). In 2019-2020, the translocation amounts of leaf, stem and sheath ^13^C photosynthates under the W3 treatment were significantly higher than that of W1, W2, W4 and W5 treatments. The translocation amounts of spike axis and husk under W3 treatment was significantly higher than that of W1 treatment, and there was no significant difference between W2, W4, and W5 treatments. Collectively, these results indicated that the W3 treatment substantially promoted the translocation of photosynthates to the grain, especially from the leaf, stem and sheath.

### 3.4 The endogenous hormone of grain after anthesis

**Figure 6:**
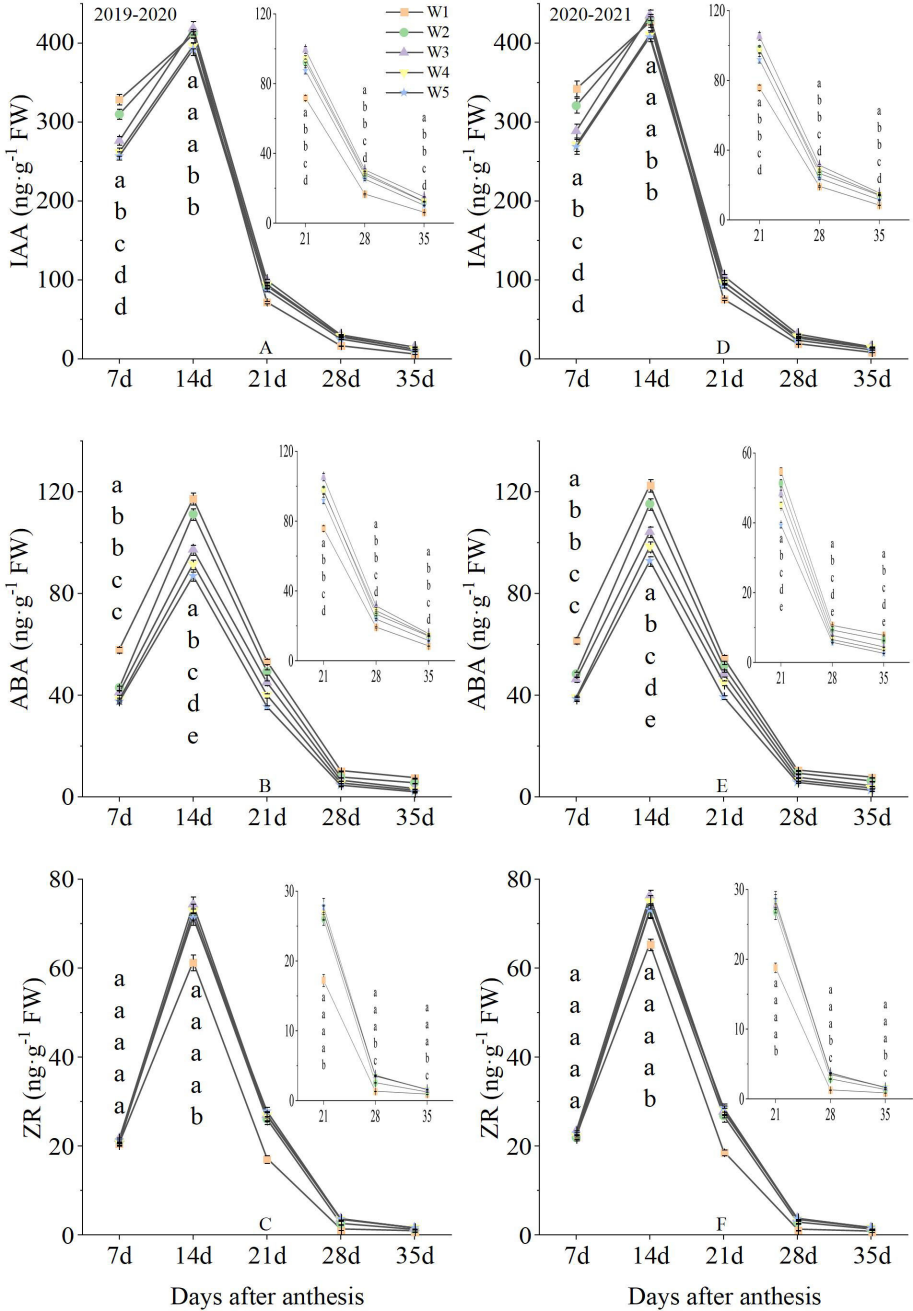
Grain filling rate after anthesis under different treatments (g·d^-1^). Lowercase letters represent that the data difference between processing is greater than 0.05.

Fluctuations in endogenous hormone of grain were observed under different treatment conditions over the two-season study (**Figure 6**). The IAA content at 7 day under W1 treatment was significantly higher than that of W2, W3, W4 and W5 treatments. W1, W2 and W3 treatments produced the highest levels at 14 day, with W4 and W5 treatments producing the lowest levels. The IAA content from 21 to 35 day under W3 treatment was significantly higher than that of W1, W2, W4 and W5 treatments. Over the 2-year measured period, ABA content after anthesis significantly decreased with increasing irrigation levels. The ZR content was not significantly different between treatments at 7 day. The ZR content from 14 to 21 day under W2, W3, W4 and W5 treatments were significantly higher than that of W1 treatment. And the W3, W4 and W5 treatments produced the highest levels from 28 to 25 day, followed by W2, with W1 producing the lowest levels. The two growing seasons had similar results, suggesting that endogenous hormone of grain at the middle and late growing stages was improved by W3 treatment.

### 3.5 1000-grain weight

**Figure 7:**
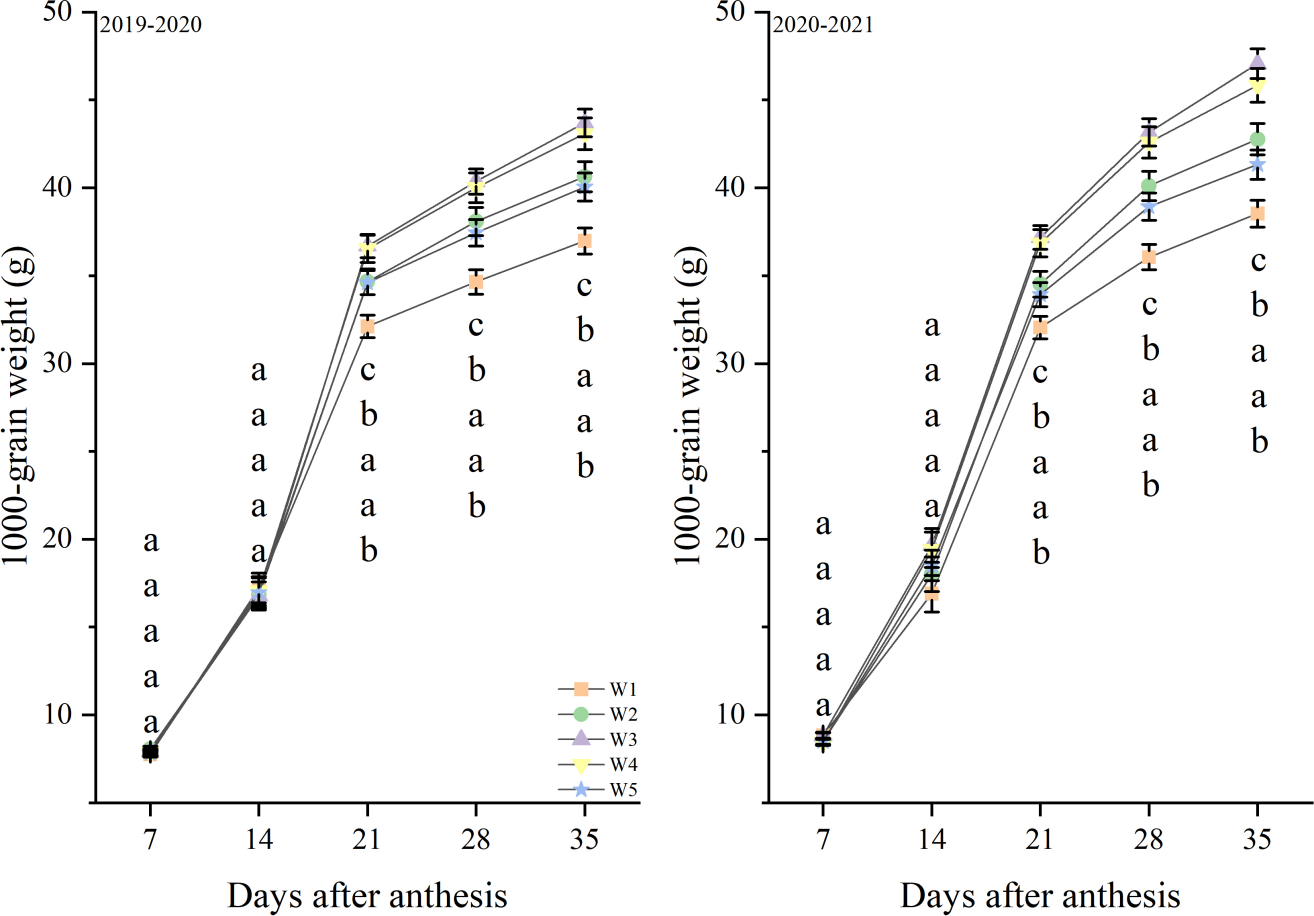
1000-grain weight after anthesis under different treatments (g). Lowercase letters represent that the data difference between processing is greater than 0.05.

In the growing seasons during 2019 and 2021, the grain weight tended to increase through the days after anthesis. The grain weight were not significantly different between treatments at 7, 14 day (**Figure 7**). And the W3 and W4 treatments produced the highest levels from 21 to 35 day, followed by W2 and W5, with W1 producing the lowest levels. Similar responses to treatments were observed in both growth seasons.

### 3.6 Grain-filling rate

**Figure 8:**
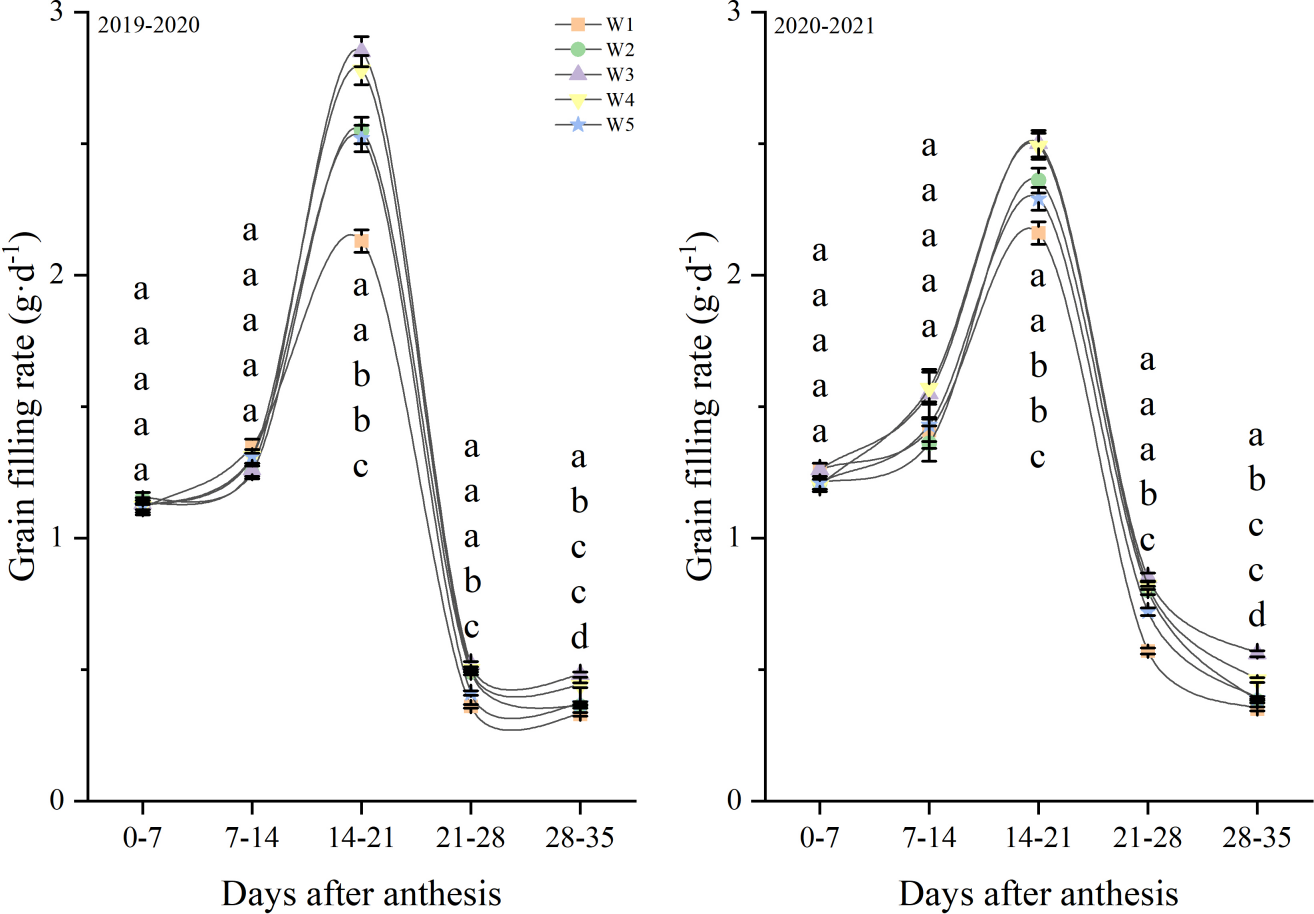
Grain filling rate after anthesis under different treatments (g·d^-1^). Lowercase letters represent that the data difference between processing is greater than 0.05.

**Figure 8** shows the changes in the grain-filling rate after anthesis. In the growing seasons during 2019 and 2021, the grain-filling rate peaked in the 14-21 days after anthesis before then decreasing. The grain-filling rate in 14-21, 21-28 and 28-35 days after anthesis were affected by the treatment, but the grain filling rate in 0-7 and 7-14 days after anthesis were no affected by the treatment. And the W3 and W4 treatments produced the highest levels from 21 to 35 day, followed by W2 and W5, with W1 producing the lowest levels. Similar responses to treatments were observed in both growth seasons.

### 3.7 Grain filling equation

The grain weight of different days after anthesis was used to carry out grouting fitting according to logistics equation (**Table 2**). In 2019-2020, the W5 treatment produced the highest the time to reach the maximum rate, followed by W2, W3 and W4, with W1 producing the lowest levels. The W3 and W4 treatments produced the highest maximum filling rate, followed by W2 and W5, with W1 producing the lowest levels. The mean filling rate and grain filling duration under the W3 treatment were significantly higher than that of W1, W2, W4 and W5 treatments. The two growing seasons had similar results, suggesting that mean filling rate and grain filling duration were improved by W3 treatment.

**Table 1:** Effects of different treatments on ^13^C distribution amount in different organs at 72h after labeling and maturity in wheat plants.

	Year	Treatments	13C distribution amount (ug·stem-1)			
			leaf	Stem and sheath	Spike axis and husk	Grain
At 72 h after labeling	2019-2020	W1	60.01b	222.65b	108.07a	–
		W2	64.14a	237.63a	113.00a	–
		W3	66.86a	260.72a	114.44a	–
		W4	68.16a	249.80a	118.44a	–
		W5	68.42a	242.35a	121.40a	–
	2020-2021	W1	72.30b	287.82b	120.39a	–
		W2	79.39a	307.61a	123.22a	–
		W3	82.80a	329.09a	128.48a	–
		W4	82.18a	314.89a	126.91a	–
		W5	83.06a	305.76a	136.39a	–
Year			**	***	***	–
Treatments			***	***	***	–
Year×Treatments			ns	ns	ns	–
At maturity	2019-2020	W1	28.00c	65.06c	42.25b	247.39c
		W2	29.09c	65.06c	43.47b	278.71b
		W3	27.54c	67.11b	44.36b	298.80a
		W4	30.90b	66.36b	45.83b	295.50a
		W5	33.24a	69.15a	47.98a	274.98b
	2020-2021	W1	27.41c	86.78c	48.94b	282.18c
		W2	30.50c	86.90c	48.38b	313.97b
		W3	29.37c	86.01b	53.28b	331.28a
		W4	31.36b	84.69b	50.91b	327.54a
		W5	34.80a	88.13a	56.96a	307.97b
Year			***	***	***	***
Treatments			***	*	***	***
Year×Treatments			*	ns	**	ns

Different letters in the same column meant significant difference at 0.05 level. *: P<0.05; **: P<0.01; ***: P<0.001.

**Table 2:** Grain filling equation and grain-filling parameters for different treatments.

Treatments		Growth curve equation	Tmax (d)	Vmax (mg·grain^-1^·d^-1^)	Vmean (mg·grain^-1^·d^-1^)	D (d)
2019-2020	W1	y=38.4437/(1 + 20.2076e-0.2093x)	14.16c	2.01c	1.05d	24.79c
	W2	y=41.4700/(1 + 24.7534e-0.2156x)	15.33b	2.17b	1.13c	27.83b
	W3	y=43.9860/(1 + 31.2705e-0.2259x)	15.24b	2.48a	1.30a	28.56a
	W4	y=43.3752/(1 + 30.9228e-0.2279x)	15.06b	2.47a	1.22b	27.32b
	W5	y=40.6048/(1 + 27.1554e-0.2254x)	16.16a	2.26b	1.13c	27.96b
2020-2021	W1	y=39.4664/(1 + 15.9270e-0.1899x)	14.38c	1.87c	1.01d	27.44c
	W2	y=43.7319/(1 + 19.7233e-0.1971x)	15.13b	2.16b	1.13c	30.14b
	W3	y=47.8077/(1 + 19.6005e-0.1943x)	15.31b	2.36a	1.32a	31.88a
	W4	y=46.4992/(1 + 21.5542e-0.2029x)	15.13b	2.32a	1.24b	29.56b
	W5	y=42.1346/(1 + 18.2619e-0.1982x)	16.25a	2.09b	1.10c	30.07b

Tmax, the time to reach the maximum filling rate; Vmax, maximum filling rate; Vmean, mean filling rate; D, grain filling duration.

### 3.8 Yield and yield components

Compared to W1, W2 and W5 treatments, W3 and W4 treatments significantly increased the grain number per spike, 1000-grain weight and grain yield, and from the perspective of water saving, the effect of W3 treatment was the highest (**Table 3**). In comparison to W1, W2 and W5 treatment, W3treatment significantly increased the two-season average grain yield by 19.69%, 6.30% and 8.07%, respectively. However, the grain yield showed no difference between W3 and W4. Except for the spike number, in comparison to W3 treatment, W5 treatment markedly decreased the two-season average grain number per spike and 1000-grain weight by 11.32% and 11.52%. The differences in Spike number, Grain number, 1000-grain and Grain yield among treatments were extremely significant, and the differences between years were extremely significant, while the differences in Year×Treatments were not significant.

**Table 3:** Grain yield and yield components under the different treatments during 2019-2021.

Year	Treatment	Spike number	Grain number	1000-grain	Grain yield
		(×10^4^·hm^-2^)	per spike	weight (g)	(kg·hm^-2^)
2019	W1	602.40b	33.03c	38.47c	7726.80c
–	W2	639.88a	35.42b	41.57b	8705.10b
2020	W3	642.73a	38.39a	44.25a	9333.90a
	W4	650.93a	37.35a	43.93a	9229.40a
	W5	660.61a	34.81b	40.56b	8588.70b
2020	W1	617.11b	34.66c	39.03c	8713.56c
–	W2	653.73a	37.36b	43.31b	9806.31b
2021	W3	660.76a	40.99a	47.65a	10343.60a
	W4	671.14a	39.41a	46.41a	10230.23a
	W5	682.72a	36.50b	41.85b	9619.27b
Year		**	***	***	***
Treatments		***	***	***	***
Year×Treatments		ns	ns	ns	ns

Different letters in the same column meant significant difference at 0.05 level. *: P<0.05; **: P<0.01; ***: P<0.001.

## 4 Discussion

### 4.1 Effects of soil moisture on grain yield and yield components

Soil water deficits lead to reduced wheat grain yield, and supplementary irrigation can increase wheat grain yield. In our study, grain yields were lowest in the W1 (low-irrigation) treatment and tended to increase with increasing irrigation level, in agreement with previous reports. The micro-sprinkler water-saving irrigation has been shown to be an effective wheat water-saving irrigation method. In our study, grain yield were higher in the W3 treatment than in other irrigation treatments. We attribute the increase in grain yield to the increase in grain number per spike and 1000-grain weight. Furthermore, our experimental field results revealed that in the W4 treatment, irrigation water use was increased by 6.67% (compared to the W3 treatment) but grain number per spike, 1000-grain weight and grain yield did not decrease significantly. However, the soil water content continued to increase to the 85% treatment level (W5), and grain number per spike, 1000-grain weight and grain yield decreased significantly (compared to the W3 treatment). The spike number of wheat increases with an increase in the soil water level supply, but the grain number per spike, 1000-grain weight and grain yield increases with soil water level act to a certain level and then declines. An soil water level in 75% maintains grain yield at relatively high levels, and further increasing the level has no benefit to increasing grain yields.

### 4.2 Effects of soil moisture on root morphological pattern

The growth and development of wheat root system have strong plasticity, and can adjust their morphological structure and spatial distribution to adapt to the environment according to soil moisture (Yang et al., 2018). It is of great significance to ensure high grain yield by adopting appropriate irrigation measures to form environmental conditions that are conducive to increasing the proportion of deep roots (Miao et al., 2019). Many researchers believe that root dry weight density and surface area density are important target for studying root distribution (Fang et al., 2021; Chen et al., 2020). The micro-sprinkler water-saving irrigation affected the infiltration and redistribution of water in soil, which affected the size and distribution of roots (Ma et al., 2013). This study showed that with the increase in soil moisture level, the root dry weight density and surface area density increased, while the proportion of root dry weight density and surface area density in deep soil depths decreased, which is because wheat roots do not need to explore deep soil depths under sufficient water conditions. Excessive soil moisture content significantly promoted root system in 0-40 cm soil, while the root dry weight density and surface area density in 40-60 cm soil depth under suitable soil moisture content were significantly greater than those under other soil moisture. Increasing deep roots helps make full use of water storage in deep soil depths (Wang et al., 2014; Thapa et al., 2019), thus reducing the risk of yield loss due to drought. Similar observation also showed that compared with W2, W3, W4 and W5 treatments, wheat under W1 had lower root dry weight density and surface area density in 0-60 cm soil depth (Jha et al., 2017). A suitable soil moisture content optimized the distribution of roots in different soil depths, coordinating the proportion of shallow and deep soil roots. Root dry weight density and surface area density are often used to assess the effects of different environmental and management factors on root distribution patterns (Ahmadi et al., 2018; Zhang et al., 2020). Based on observations of root dry weight density and surface area density, our study showed that suitable soil moisture content optimized the root morphological pattern of wheat by increasing root dry weight density and surface area density of deep roots.

### 4.3 Effects of soil moisture on accumulation and transport of photosynthetic products

In order to explain the soil moisture of W3 on accumulation, transport and distribution of photocontracides, we use ^13^C isotope tracer technique to investigate. Plants need to allocate more dry matter to vegetative organs compounds to maximize the productivity of water (Feng et al., 2023). In this study, the amount of ^13^C photosynthate in vegetative organs at 72h after labeling increased with the increase of soil moisture level, but there was no significant difference between W2, W3, W4, and W5 treatments. As the photosynthetic material production exceeds the respiratory consumption, the excess photosynthetic product is stored in vegetative organs as dry matter, which is transported after anthesis to meet the needs of grain filling (Zhao et al., 2020). In this study, compared with other soil water level treatments, W3 increased the ^13^C photosynthate in grain at maturity, simultaneously. High soil water level (W5) further increased the ^13^C photosynthate at maturity but most of those increased ^13^C photosynthate was allocated to the vegetative organs pools instead of the grain pool, which explained the decrease in grain yield under W5 treatment. Compared with the W1, W2, W4 and W5 treatments, the translocation amounts of leaf, spike axis and husk under the W3 treatment had significantly higher pre-anthesis ^13^C photosynthate. In this experiment, the W3 treatment tended to allocate more ^13^C photosynthate to grains and less to vegetative organs, compared with other treatments, which explained why W3 had higher ^13^C photosynthate in grain than other treatments.

### 4.4 Grain-filling in wheat grain is regulated by plant hormones

Water is an essential condition for plant growth, development, and production. The hormones play key roles during plant growth and development and in response to environmental factors and water deficit. In this study, we found that both IAA and zeatin riboside (ZR) were significantly reduced by excessive low or high soil moisture level after anthesis in grain. However, with the increase of soil moisture level, the content of ABA in grain decreased gradually. These results indicated that increasing soil moisture content could significantly delay plant senescence. The relationship between hormones and irrigation management has also been intensively studied in vegetative organs. In tea plants, Drought stress was found to reduce cytokinin content by inhibiting the biosynthesis of ZR and IAA (Hu et al., 2020). In rice, the application of irrigation increased the expression of ZR and IAA biosynthesis genes and thus promoted ZR and IAA content in roots and leaves (Zhu et al., 2018). However, under excessive irrigation conditions, a high level of ZR and IAA will lead to “staygreen” and delayed whole plant senescence (Gregersen et al., 2013). In this study, IAA was inhibited by high or low soil moisture level, suggesting that inhibition of IAA biosynthesis by high or low occurs in grains. In addition, low soil moisture level also suppressed the content of ZR, But high soil moisture level did not significantly promote grain ZR content. These results suggest that ZR content might was regulated by catabolism rather than biosynthesis in response to high soil moisture level treatment. Under moderate water stress, ABA establishes and maintains root meristem function and stimulates root elongation, and ABA response and auxin transport play key roles in root elongation under water stress (Li et al., 2024). Under drought conditions, local accumulation of root auxin is consistent with its role in inhibiting root aquaporins and reducing vascular cell size, limiting vascular diameter and limiting root radial aquaporin-mediated water transport (Walid and Rémy, 2019).

The grain-filling process is the conversion between dry matter that involves photoassimilate translocation and accumulation in grains. Previous studies have shown that a low irrigation amount leads to premature senescence and (Zhang et al., 2016) reducing the grain-filling time (Wang et al., 2009). However, irrigation can be expected to postpone the premature senescence of plants and prolong the grain-filling time (Yang and Zhang, 2010). The grain-filling rate and grain-filling time are critical parameter affecting wheat grain weight. In our study, the grain filling was inhibited by high or low soil moisture, and the grainfilling rate and grain weight showed similar trends. And the grain filling parameters results indicate that the inhibitory effect of high or low soil moisture on grain weight is caused mainly by the reduction in the grain-filling rate and grain-filling time. In agreement with a previous finding (Hamner et al., 2017), high soil moisture markedly delayed the time to reach the maximum filling rate. Balotf et al. (2018) stated that irrigation promotes the synthesis of carbohydrates and their transport to the grain by promoting photosynthesis. However, Liang et al. (2017) showed that the amount of carbohydrates transported to the grain decreased under high soil moisture conditions, owing to the large amount of carbohydrates consumed for the vigorous growth of vegetative organs such as stems and leaves under high soil moisture conditions (Sun et al., 2019). Thus, the effects of irrigation on carbohydrate transport can vary. In the present study, high soil moisture delayed the time to reach the maximum filling rate. However, high soil moisture also promotes the growth of vegetative organs such as stems, and this consumes many carbohydrates and may reduce their transport to the grain.

## 5 Conclusion

This study reported comprehensive measurements from winter wheat fields under five micro-sprinkler water-saving irrigation practices (i.e. W1, W2, W3, W4 and W5) in the North China Plain during 2019–2021. The results showed that under the condition of micro-spray water-saving irrigation, W3 treatment promoted the development of wheat roots after anthesis in deep soil (0-60 cm). At the same time, W3 promoted the transfer of photosynthetic products from leaves, stems and sheaths to grains by increasing endogenous hormones in grains, and improved the average filling rate and grain filling time, and finally increased the yield through carbon redistribution. The study revealed the mechanism of optimizing micro-sprinkler water-saving irrigation for improving grain yield and provided a new perspective for improve wheat grain yield. In future experiment, endogenous hormone of leaf and photosynthetic rate can be observed simultaneously, so as to quantify the effect of micro-sprinkler water-saving irrigation on photosynthetic material production and provide more sufficient evidence for the results. The results also implied that ensuring water supply throughout the winter wheat growth period, especially during critical growth periods, could mitigate the negative effects of water scarcity. Whether the shift in irrigation modes will have a positive impact on other crops in future climate scenarios requires further exploration.

## Data Availability

The original contributions presented in the study are included in the article/supplementary material, further inquiries can be directed to the corresponding author/s.

## References

[B1] Ahmadi S. H. Sepaskhah A. R. Zarei M. (2018). Specific root length, soil water status, and grain yields of irrigated and rainfed winter barley in the raised bed and flat planting systems. Agric. Water Manage. 210, 304–315. doi: 10.1016/j.agwat.2018.08.031

[B2] Balotf S. Islam S. Kavoosi G. Kholdebarin B. Juhasz A. Ma W. (2018). How exogenous nitric oxide regulates nitrogen assimilation in wheat seedling under different nitrogen source and levels. PloS One 12, e0190269. doi: 10.1371/journal.pone.0190269 PMC5761883 29320529

[B3] Chang S. Chen Y. Jia S. Li Y. Liu K. Lin Z.. (2020). Auxin apical dominance governed by the OsAsp1-OsTIF1 complex determines distinctive rice caryopses development on different branches. PloS Genet. 16, e1009157. doi: 10.1371/journal.pgen.1009157 33108367 PMC7647119

[B4] Chen Y. L. Palta J. Prasad P. V. V. Siddique K. H. M. (2020). Phenotypic variability in bread wheat root systems at the early vegetative stage. BMC Plant Biol. 20, 185. doi: 10.1186/s12870-020-02390-8 32345227 PMC7189723

[B5] Fang F. Liang Liu S. Xu B. C. Siddique K. Palta J. A.. (2021). Wheat cultivars with small root length density in the topsoil increased post-anthesis water use and grain yield in the semi-arid region on the Loess Plateau. Eur. J. Agron. 124, 1–13. doi: 10.1016/j.eja.2021.126243

[B6] Feng S. Ding W. Shi C. Zhu X. Hu T. Ru Z. (2023). Optimizing the distribution of roots by supplemental irrigation to improve grain yield and water use efficiency of wheat in the North China Plain. Agric. Water Manage. 275, 107989. doi: 10.1016/j.agwat.2022.107989

[B7] Gao J. Zhao B. Dong S. Liu P. Ren B. Zhang J. (2017). Response of summer maize photosynthate accumulation and distribution to shading stress assessed by using ^13^CO_2_ stable isotope tracer in the field. Front. Plant Sci. 8, 1821. doi: 10.3389/fpls.2017.01821 29123536 PMC5662628

[B8] Gleason S. Cooper M. Wiggans D. Bliss C. Romay M. Gore M.. (2019). Stomatal conductance, xylem water transport, and root traits underpin improved performance under drought and well-watered conditions across a diverse panel of maize inbred lines. Field Crop Res. 234, 119–128. doi: 10.1016/j.fcr.2019.02.001

[B9] Gregersen P. Culetic A. Boshian L. Krupinska K. (2013). Plant senescence and crop productivity. Plant Mol. Biol. 82, 603–622. doi: 10.1007/s11103-013-0013-8 23354836

[B10] Hamner K. Weih M. Eriksson J. Kirchmann H. (2017). Influence of nitrogen supply on macro- and micronutrient accumulation during growth of winter wheat. Field Crop Res. 213, 118–129. doi: 10.1016/j.fcr.2017.08.002

[B11] Hu S. Zhang M. Yang Y. Xuan W. Zou Z. Arkorful E.. (2020). A novel insight into nitrogen and auxin signaling in lateral root formation in tea plant [Camellia sinensis (L.) O. Kuntze. BMC Plant Biol. 20, 232. doi: 10.1186/s12870-020-02448-7 32448156 PMC7247184

[B12] Jha S. K. Gao Y. Liu H. Huang Z. Wang G. Liang Y.. (2017). Root development and water uptake in winter wheat under different irrigation methods and scheduling for North China. Agric. Water Manage. 182, 139–150. doi: 10.1016/j.agwat.2016.12.015

[B13] Kang S. Hao X. Du T. Tong L. Su X. Lu H.. (2017). Improving agricultural water productivity to ensure food security in China under changing environment: from research to practice. Agric. Water Manage. 179, 5–17. doi: 10.1016/j.agwat.2016.05.007

[B14] Li Y. Jiang S. Q. Hong Y. H. Yao Z. X. Chen Y. D. Zhu M.. (2024). Transcriptomic and hormonal changes in wheat roots enhance growth under moderate soil drying. Int. J. Mol. Sci. 25, 9157. doi: 10.3390/ijms25179157 39273103 PMC11395032

[B15] Li J. Zhang Z. Liu Y. Yao C. Song W. Xu X.. (2019). Effects of micro-sprinkling with different irrigation amount on grain yield and water use efficiency of winter wheat in the North Chin Plain. Agric. Water Manage. 224, 105736. doi: 10.1016/j.agwat.2019.105736

[B16] Liang W. Zhang Z. Wen X. Liao Y. Liu Y. (2017). Effect of non structural carbohydrate accumulation in the stem pre-anthesis on grain filling of wheat inferior grain. Field Crop Res. 211, 66–76. doi: 10.1016/j.fcr.2017.06.016

[B17] Liu Y. Han M. Zhou X. Li W. Du C. Zhang Y.. (2022). Optimizing nitrogen fertilizer application under reduced irrigation strategies for winter wheat of the North China Plain. Irri Sci. 40, 255–265. doi: 10.1007/s00271-021-00764-w

[B18] Liu Y. Liao Y. Liu W. (2021). High nitrogen application rate and planting density reduce wheat grain yield by reducing filling rate of inferior grain in middle spikelets. Crop J. 9, 412–426. doi: 10.1016/j.cj.2020.06.013

[B19] Ma L. H. Liu X. L. Wang Y. K. Wu P. T. (2013). Effects of drip irrigation on deep root distribution, rooting depth, and soil water profile of jujube in a semiarid region. Plant Soil. 37, 995–1006. doi: 10.1007/s11104-013-1880-0

[B20] Man J. G. Wang D. White P. J. Yu Z. W. (2014a). The length of micro-sprinkling hoses delivering supplemental irrigation affects photosynthesis and dry matter production of winter wheat. Field Crop Res. 168, 65–74. doi: 10.1016/j.fcr.2014.08.012

[B21] Man J. G. Yu J. S. White P. J. Gu S. B. Zhang Y. L. Guo Q. F.. (2014b). Effects of supplemental irrigation with micro-sprinkling hoses on water distribution in soil and grain yield of winter wheat. Field Crop Res. 161, 26–37. doi: 10.1016/j.fcr.2014.02.001

[B22] Miao Q. X. Fang Y. Chen Y. L. (2019). Studies in the responses of wheat root traits to drought stress. Chin. Bull. Bot. 54, 652–661. doi: 10.11983/CBB19089

[B23] Nonhebel H. M. Griffin K. (2020). Production and roles of IAA and ABA during development of superior and inferior rice grains. Funct. Plant Biol. 47, 716–726. doi: 10.1071/FP19291 32438973

[B24] Panda B. B. Sekhar S. Dash S. K. Behera L. Shaw B. P. (2018). Biochemical and molecular characterisation of exogenous cytokinin application on grain filling in rice. BMC Plant Biol. 18, 1–19. doi: 10.1186/s12870-018-1279-4 29783938 PMC5963110

[B25] Sun Z. Wu S. Zhang Y. Meng F. Zhu B. Chen Q. (2019). Effects of nitrogen fertilization on pot-grown wheat photosynthate partitioning within intensively farmed soil determined by ^13^C pulse-labeling. J. Plant Nutr. Soil Sci. 182, 896–907. doi: 10.1002/jpln.201800603

[B26] Sun H. Zhang X. Wang E. Chen S. Shao L. (2015). Quantifying the impact of irrigation on groundwater reserve and crop production-a case study in the North China Plain. Eur. J. Agron. 70, 48–56. doi: 10.1016/j.eja.2015.07.001

[B27] Teng Z. N. Yu H. H. Wang G. Q. Meng S. Liu B. H. Yi Y. K.. (2021). Synergistic interaction between ABA and IAA due to moderate soil drying promotes grain filling of inferior spikelets in rice. Plant J. 109, 1457–1472. doi: 10.1111/tpj.v109.6 34921476

[B28] Thapa S. Xue Q. Jessup K. E. Rudd J. C. Baker S. (2019). Yield determination in winter wheat under different water regimes. Field Crop Res. 233, 80–87. doi: 10.1016/j.fcr.2018.12.018

[B29] Walid S. Rémy S. (2019). Potential involvement of root auxins in drought tolerance by modulating nocturnal and daytime water use in wheat. Ann. Bot. 124, 969–978. doi: 10.1093/aob/mcz023 30918962 PMC6881217

[B30] Wan W. Zhao Y. Wang Z. Li L. Jing J. Lv Z.. (2022). Mitigation fluctuations of inter-row water use efficiency of spring wheat via narrowing row space in enlarged lateral space drip irrigation systems. Agric. Water Manage. 274, 107958. doi: 10.1016/j.agwat.2022.107958

[B31] Wang C. Y. Liu W. X. Li Q. X. Ma D. Y. Lu H. F. Feng W.. (2014). Effects of different irrigation and nitrogen regimes on root growth and its correlation with above-ground plant parts in high-yielding wheat under field conditions. Field Crop Res. 165, 138–149. doi: 10.1016/j.fcr.2014.04.011

[B32] Wang R. Liu G. S. Ni G. S. Bi Q. W. Yang L. B. Zhen C, H. (2009). Effects of planting density on photosynthetic characteristics and assimilate accumulation of leaves in different positions in Flue-Curred Tobacco.. Crop J. 12, 2288–2295.

[B33] Wang Z. Xu Y. Chen T. Zhang H. Yang J. Zhang J. (2015). Abscisic acid and the key enzymes and genes in sucrose-to-starch conversion in rice spikelets in response to soil drying during grain filling. Planta 241, 1091–1107. doi: 10.1007/s00425-015-2245-0 25589060

[B34] Wu Q. Wang Y. Chen T. Zheng J. Sun Y. Chi D. (2020). Soil nitrogen regulation using clinoptilolite for grain filling and grain quality improvements in rice. Soil Tillage Res. 199, 104547. doi: 10.1016/j.still.2019.104547

[B35] Yang B. Wang P. Y. You D. B. Liu W. J. (2018). Coupling evapotranspiration partitioning with root water uptake to identify the water consumption characteristics of winter wheat: A case study in the North China Plain. Agric. For. Meteorol. 259, 296–304. doi: 10.1016/j.agrformet.2018.05.017

[B36] Yang J. Zhang J. (2010). Grain-filling problem in super rice. J. Exp. Bot. 61, 1–5. doi: 10.1093/jxb/erp348 19959608

[B37] Yu Z. Duan X. Luo L. Dai S. Ding Z. Xia G. (2020). How plant hormones mediate salt stress responses. Trends Plant Sci. 25, 1117–1130. doi: 10.1016/j.tplants.2020.06.008 32675014

[B38] Zhang Y. Dai X. Jia D. Li H. Wang Y. Li C.. (2016). Effects of plant density on grain yield, protein size distribution, and breadmaking quality of winter wheat grown under two nitrogen fertilisation rates. Eur. J. Agron. 73, 1–10. doi: 10.1016/j.eja.2015.11.015

[B39] Zhang X. X. Whalley P. A. Ashton R. W. Evans J. Hawkesford M. J. Griffiths S.. (2020). A comparison between water uptake and root length density in winter wheat: effects of root density and rhizosphere properties. Plant Soil. 451, 345–356. doi: 10.1007/s11104-020-04530-3 32848280 PMC7437669

[B40] Zhang Z. Yu Z. W. Shi Y. Zhang Y. L. (2023). Effects of micro-sprinkling with different irrigation levels on winter wheat grain yield and greenhouse gas emissions in the North China Plain. Eur. J. Agron. 143, 126725. doi: 10.1016/j.eja.2022.126725

[B41] Zhao H. Zhang P. Wang U. Ning T. Xu C. Wang P. (2020). Canopy morphological changes and water use efficiency in winter wheat under different irrigation treatments. J. Inter Agr. 19, 1105–1116. doi: 10.1016/S2095-3119(19)62750-4

[B42] Zhu K. Zhan M. Chen J. Wang Z. Yang J. Zhao B. (2018). Effects of irrigation regimes during grain filling under different nitrogen rates on inferior spikelets grain-filling and grain yield of rice. Chin. J. Rice Sci. 32, 155–168. doi: 10.16819/j.1001-7216.2018.7060

